# The Relationship between Health Status and Social Activity of Perimenopausal and Postmenopausal Women (Health Status and Social Relationships in Menopause)

**DOI:** 10.3390/ijerph17228388

**Published:** 2020-11-12

**Authors:** Beata Naworska, Anna Brzęk, Monika Bąk-Sosnowska

**Affiliations:** 1 Department of Perinatology and Oncological Gynecology, Faculty of Health Sciences in Katowice, Medical University of Silesia in Katowice, Medyków 12 Str., 40-752 Katowice, Poland; bnaworska@sum.edu.pl; 2 Department of Physiotherapy, Chair of Physiotherapy, Faculty of Health Sciences in Katowice, Medical University of Silesia in Katowice, Medyków 12 Str., 40-752 Katowice, Poland; 3 Department of Psychology, Chair of Social Sciences and Humanities, Faculty of Health Sciences in Katowice, Medical University of Silesia in Katowice, Medyków 12 Str., 40-752 Katowice, Poland; monika.bak-sosnowska@sum.edu.pl

**Keywords:** perimenopause, postmenopause, climacteric syndrome, depression, physical activity

## Abstract

The quantity and quality of interpersonal relations (including participation in University of the Third Age—U3A) play an important role for women during menopausal changes. Women who have a social network are found to be more positive about menopause, and are less likely to be depressed. This case-control study aimed to analyze the relationship between participating in formal social groups and health status related to physical activity and climacteric and depressive symptoms. The study was conducted among 621 peri- and postmenopausal women aged 50–64 years. The women were classified into two groups: U3A and controls. The participants were selected using a multistage sampling method. The IPAQ (The International Physical Activity Questionnaire), Kupperman Index (KI), and Beck Depression Inventory were used for data collection. Significant differences between the groups were confirmed in the area of professional work (*p* < 0.001), free time (*p* < 0.001), and sitting (*p* < 0.05). The average KI score in the U3A group was higher (t-Student = 2.12, *p* < 0.05). Depressive symptoms were found in 43.49% of U3A women vs. 51.15% in controls (*p* < 0.01). We conclude that participation in formal social groups is associated with higher level of physical activity and reduced severity of both climacteric and depressive symptoms.

## 1. Introduction

The World Health Organization (WHO) defines menopause as the last normal monthly period of bleeding in a woman’s life, which is causally linked to the total loss of ovarian follicles. According to the menopausal division of a woman’s life, there are three consecutive periods adjacent to menopause: menopausal transition period, perimenopause, and postmenopause. Premenopause is defined as the stage before the last menstrual period in which the irregularity of menstruation is usually increased and falls in the age range of about 45–49 years. Perimenopause includes the period immediately before menopause (about two years), when the first clinical, biological, and endocrine features of the approaching last menstrual period begin to appear, as well as one to two years after menopause. Postmenopause, on the other hand, is defined by the WHO as the period of life after the last menstrual period, regardless of whether menopause is natural or artificially induced [1]. In Polish conditions, it is assumed that the last menstruation (menopause) occurs most often around 49.3 years of age [2].

The period of menopausal transition may be significantly impaired by some ailments and discomforts that deteriorate the quality of life. Most women experience vasomotor symptoms such as hot flushes or night sweats, and some also experience sleep and mood disorders, headaches, problems with concentration and memory, arrhythmia, joint pain, atrophic vaginitis, irritability, and general malaise [3]. Some women may also suffer from depressive disorders, especially when accompanied with severe vasomotor symptoms [4]. Even though hormone therapy (HT) is an effective method of relieving menopausal symptoms [5], it is unsuitable for some women because of medical contraindications. Others, according to personal preference, choose nonpharmacological methods of alleviating ailments, such as phytoestrogens (PEs) [6].

There are some discrepancies in categorizing the risk factors for depression in the menopausal period. Some authors believe that the climacteric period alone is a risk factor for discouraged mood and symptoms of depression in women without a history of depression [7]. The other authors postulate that women with an earlier onset of menopause, as well as those with previous negative life experiences, are more likely to have depression. Moreover, a medical history of depression or somatic problems may affect the severity of the climacteric syndrome [8].

The quantity and quality of interpersonal relationships play an important role for women during menopausal changes and may affect both mental and physical health, quality of life, and mortality risk. Adults who are more socially connected are healthier, live longer, and have a better quality of life than their more isolated peers [9,10].

Additionally, a negative psychosocial environment is a factor that favors the development of psychological and vasomotor symptoms during the period of menopausal transition [11]. Women who have a social network are found to be more positive about menopause and are less likely to be depressed [12]. In contrast, it was proven that deficits and more negative social interactions are associated with higher incidence of depression. Such a phenomenon is more common in women, as they are more likely than men to experience some life events reducing social relations: widowhood, illness, and financial strain [13].

Research on the relationship between social life and the healthiness of middle-aged women is usually based on informal relationships with the spouse (which is perhaps the most often studied social relation), children, and friends [14]. Definitely less research has been conducted on formal relationships, especially those intentionally selected as an alternative to informal relations. Thus, the subject of our innovative research was dedicated to women who attended the University of the Third Age (U3A) and their formalized social relations. Such didactic institutions have been established in many countries as branches of universities or community associations, offering seniors classes on science, art, movement, and other topics. Classes provide a stimulus to mobilize the participants physically and intellectually, as well as giving an opportunity to establish new interpersonal relationships due to regular contact between the participants who share information about specific activities, spend time together, and create and strengthen health behaviors. In Poland, the minimum age for participation in U3A is 50, which is a perimenopausal period for most women. The results of the research show that participation in the activities of the University of the Third Age has a positive impact on health and quality of life [15,16,17,18].

To the best of our knowledge, based on a PubMed data search between 1990 and 2018, there are hardly any surveys regarding the multifaceted approach, covering both the physical and mental aspects of health status of women participating in U3A. In the literature review, there are studies on the psychophysical problems of middle-aged women which are related to or are independent of menopause [19,20,21,22]. The available data also focus on selected areas of functioning in this study group [15,16,17,18]. Thus, the aim of our study was to analyze the possible relationship between physical well-being, physical activity, and mood in peri- and postmenopausal attendees of U3A.

The following research questions were formulated:

(1) Are there differences in the level of physical activity between the participants of U3A and the group not participating in any formal groups and possibly to what extent?
(2) Are there differences in the number of climacteric symptoms between the examined groups?
(3) What is the level of depression in the examined groups and are there differences between the examined groups?
(4) Is there a relationship between the level of physical activity and depression?

## 2. Materials and Methods

### 2.1. Material Status

A total of 975 women living in Silesia (Poland) were involved in the study. The inclusion criteria for the study group (A group) included: age of 50–64 years, regular participation for a minimum of three months in the activities of the University of the Third Age (U3A), conscious and voluntary consent to participate in the study. The exclusion criteria included participation in formalized social relationships (e.g., religious or other social groups) and current treatment due to depression, as well as the presence of heart failure, exacerbation of coronary heart disease, uncontrolled arterial hypertension, decompensated diabetes, untreated thyroid disease, or neoplastic changes.

The activity of the U3A is based on the functioning of organized groups of several dozen people. In Silesia, the only criterion for joining U3A is the minimum age (50 years). The groups meet regularly throughout the academic year, within the auspices of the university or as a form of local community. An educational lecture is organized at the headquarters of U3A at least once a month, but sometimes (depending on the specific U3A) additional classes are available. The additional classes are chosen by the participants according to their personal preferences among ones proposed. These activities include, for example: physical activity (Nordic walking, swimming, gymnastics, yoga), computer classes, foreign language classes, culinary classes, tourist trips, visiting museums, going to the cinemas, theaters, and concerts.

The control group (B group), recruited from the general practitioners’ practices, included women who did not participate in any formal group (except professional groups) during the previous year. Both inclusion and exclusion criteria were identical to those in the study group.

**Figure 1:**
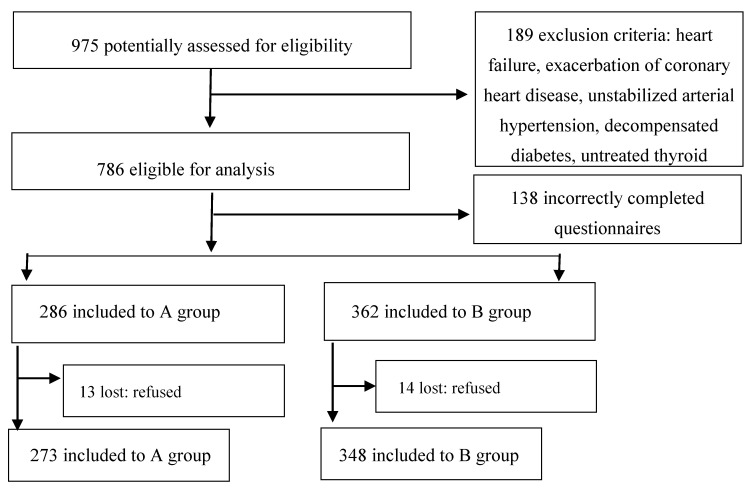
Visual insight into the participants selection procedure.

Finally, 621 women were included in the study and fully completed the questionnaires. Group A and B consisted of 273 and 348 women, respectively. The visual selection procedure of participants is shown in Figure 1.

### 2.2. Method

The observational study was designed as a case-control one. We used three standardized questionnaires.

The International Physical Activity Questionnaire (long form—IPAQ) was used to assess the level of physical activity. It contained 27 questions about activity during the last seven days related to: professional work, locomotion, housework, free time. Only activities performed for a minimum of 10 min without interruption were considered. The level of physical activity was calculated by multiplying the number of days in a week in which the given physical activity was performed by the time in minutes for a given activity. The obtained result was multiplied by an MET (metabolic equivalent of work) factor appropriate for a given activity (intense physical exercise—8 MET, moderate physical exercise—4 MET, walking—3.3 MET). Rest time was not considered. The sum of all the results for individual activities gives a weekly measurement of physical activity, presented as MET units (MET min/week). The final result falls into one of three categories defining the level of physical activity: low (<600 MET min/week), average (600–1500 MET min/week), high (>1500 MET min/week). The alpha conformity ratio for this questionnaire is estimated at 0.8 [23].

The severity of climacteric symptoms was assessed using the Kupperman Index (KI). The questionnaire is divided into 11 categories to determine the severity of symptoms on a scale ranging from a lack of any symptoms, through to light, medium, and ending with severe. For each answer, a specific number of points is assigned, the sum of which allows four levels of climacteric syndrome to be distinguished: none (0–20 points), light (21–25 points), medium (26–30 points), and severe climacteric syndrome (over 30 points).

The severity of depressive symptoms was examined using the Beck Depression Inventory (BDI), a standardized tool for assessing clinical depression and its severity. It contains 21 items, each of which consists of four statements describing the severity of a particular symptom. The subject is asked to choose one answer for each item that best describes their functioning. The overall result is obtained by adding up all the points. Obtaining 12 points or above indicates the occurrence of clinical symptoms of depression.

In addition, data regarding the educational level of the subjects, their work status, marital status, having children, material status, as well as anthropometric information (declared age, height, and weight) were collected. On this basis, the body mass index (BMI) was calculated using the following formula: BMI = body mass (kg)/height (m)^2^. The respondents also declared whether they were undertaking hormone replacement therapy or other means (parapharmaceuticals) aimed at alleviating menopausal symptoms.

### 2.3. Organization of the Study

The research was carried out both at Universities of the Third Age and in outpatient clinics in Silesia (Poland). We randomly selected every second U3A from those operating in the region. Our study was conducted at least three months after the start of U3A classes in a given academic year, during the lectures in the headquarters of the U3A. Every single woman present at the lecture was asked to participate in the study. Similarly, we randomly selected several GP (General Practices) from the list of clinics in the region. Receptionists working in the GP registration offices were asked for their cooperation. Within a selected two-week period, female patients were offered to participate in the study.

All participants were fully informed about the purpose of the study, its anonymity, and the possibility of refusing to participate in the study, and gave their consent. Subjects had the opportunity to ask questions and were provided with a copy of the information sheet.

Each subject received a set of three questionnaires to be filled out and then left either at the reception desk or in the registration office. The time for the response was not limited. Subjects were not paid for participation in the study.

### 2.4. Ethical Considerations

The research protocol was approved by the local Ethics Committee of the Medical University of Silesia in Katowice and qualified as not being a medical experiment (KNW/0022/KB/177/12). All clinical investigation was conducted according to the principles expressed in the Declaration of Helsinki.

### 2.5. Statistical Analysis

Statistical analyses were performed using the STATISTICA 14.0. Kolmogorov–Smirnov test was used to assess the normality of distributions. In the univariate analysis, dependence tests (R-Pearson, R-Spearman) and tests of significance of differences (t-Student, U-Mann–Whitney, chi-2 Pearson) were used. Statistical significance was assessed at the level of α = 0.05.

## 3. Results

The average age in group A was 59.97 ± 2.34 years, and the average BMI was 26.84 ± 3.53 kg/m^2^. In group B, mean age and BMI were 53.54 ± 3.44 years and 27.12 ± 4.38 kg/m^2^, respectively. There were no significant differences between both groups with regard to age (*p* > 0.05) and BMI (*p* > 0.05). The distribution of sociodemographic variables is presented in Table 1.

### 3.1. Physical Activity

The weekly energy expenditure in group A ranged from 118 to 25.674 MET-min/week (*x* = 3434.88 ± 4864.89), while in group B from 150 to 33315 MET-min/week (*x* = 3914.35 ± 5573.89). In group A, the highest level of physical activity was observed in the area of professional work and in group B in the area of leisure time. The details and differences between groups are presented in Table 2.

Significant differences between groups were confirmed in the area of professional work (*p <* 0.001) and free time (*p* < 0.001).

### 3.2. Climacteric Symptoms

The majority of women in group A (72.59%) did not declare the presence of climacteric symptoms, similarly to women in group B (68.79%). The details of the severity of symptoms in both groups are shown in Table 3.

The average KI score in group A was 18.31 ± 11.24, while in B 16.46 ± 9.81 points. In the studied groups, there was no correlation between the occurrence of climacteric symptoms and age (*p* > 0.05). However, a significant positive relationship was observed between the occurrence of climacteric symptoms and physical activity associated with professional work (*X*^2^ = 19.99; df = 6, *p* < 0.01) in group A, and between climacteric symptoms and physical activity associated with housework domains (*X*^2^ = 16.94; df = 6, *p* < 0.04) in group B.

### 3.3. Depressive Symptoms

**Figure 2:**
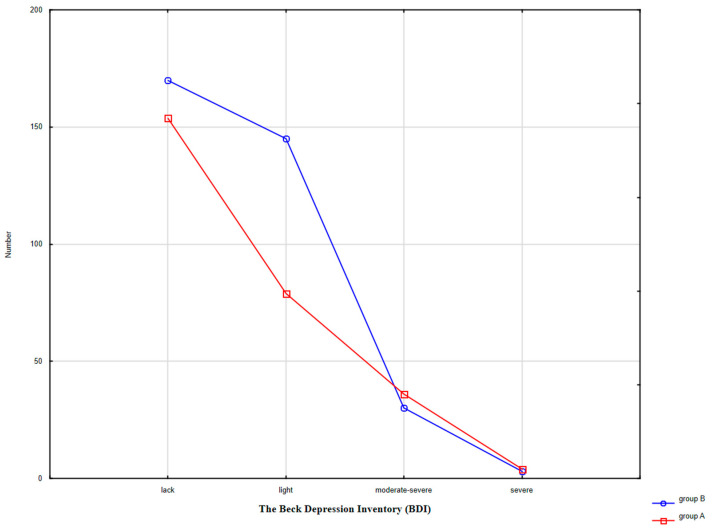
Interaction of the density of clinical symptoms of depression based on BDI (The Beck Depression Inventoty).

Depressive symptoms were found in 43.49% of women in group A and in 51.15% of women in group B. The average BDI scores were 16.77 ± 5.45 and 15.02 ± 6.03 in group A and B, respectively, which was a statistically significant difference (t-Student = 2.53, *p* < 0.01). The groups also varied in terms of the age of the respondents reporting depressive symptoms (t-Student = 17.22, *p* < 0.001). In group A, these were women aged 60.19 ± 2.22, while in group B the women were younger: 50–65 years old (*x* = 53.92 ± 3.43). Moderate and severe depressive symptoms were presented by 14.66% of women in group A and 9.48% of women in the control group (Figure 2). The most common climacteric symptoms in group A among women diagnosed with depression were: excessive sweating (76.07%), dizziness (61.54%), drowsiness (78.63%), nervousness (71.79%), lack of energy (79.49%), muscle and joint pain (57.26%), headache (63.25%), paresthesia (35.9%). In controls the results were as follows: 76.07%; 61.54%; 77.53%; 66.85%; 77.53%; 58.43%; 60.67%, and 29.78%, respectively.

There was a statistical relationship between depressive symptoms along with the severity of climacteric symptoms in both groups (for group A: *r_s_* = −0.39; *p* < 0.0001; for group B: *r_s_* = −0.28, *p* < 0.0001).

In group A, 31.6% of 72.86% of women without climacteric syndrome had no depressive symptoms. In group B, 42% of 68.97% of patients with no climacteric symptoms had no depressive symptoms as well.

A negative trend, though not statistically significant, was observed between depressive symptoms and the level of physical activity in group A (*p* > 0.16). At the same time, it was found that 54.70% and 35.90% of women diagnosed with depression were characterized by medium and high level of physical activity, respectively. In group B, the proportion was 60.45% and 28.81%, respectively. There was no relationship between physical activity and symptoms of depression (*p* > 0.27) in controls. The details are presented in Table 4.

## 4. Discussion

### 4.1. The University of the Third Age (U3A)

U3A may support preventative health measures and increase the likelihood of suppressing existing problems in the area of physical or mental health and effective treatment. Universities of the Third Age are designed to mobilize older people socially, mentally, and physically. They are an interesting proposition for women’s development and offer an active way of spending their leisure time. In the studies of Hasińska and Tracz (2013) [24] on the role of U3A in active aging, the authors pointed to the generally high level of activity among U3A students resulting from their attitudes, such as positive motivation and goal orientation, as well as having dreams and plans for the future. Over 90% of participants were interested in the world and people and liked to learn. Seniors also declared independence in thinking, self-esteem, and the ability to cope with difficulties and new situations, and felt satisfaction with their own lives [24]. There are already over 400 Universities of the Third Age in Poland [25] that are available for persons over 50 and provide them with not only educational activities, but also with a series of group activities related to specific interests.

### 4.2. Climacteric Symptoms

Menopause is a challenge for doctors and other specialists who want to improve the quality of life of their patients during that time. Women experience significant hormonal fluctuations, affecting onerous vasomotor symptoms and other physical and sexual ailments. The highest intensity of climacteric symptoms is usually observed in the age group of 56–65 years [26], which was also demonstrated in our own study, where the severe climacteric symptoms were reported by women aged between 59 and 63. Differences in observed severity of climacteric symptoms were dependent on whether the women attended or did not attend the U3A classes. More symptoms were experienced by participants in group B.

The use of menopausal hormone therapy (MHT) significantly correlates with less severe climacteric symptoms [27], which was not confirmed in our study. Regardless of participation in U3A classes, subjects using MHT were in a minority. The results of our research showed that the majority of women from both groups did not manifest climacteric symptoms despite being 50–64 years old. Similar results in the Polish population, based on the Kupperman Index, were observed by Skrzypulec et al. The authors indicated generally low mean index values [2]. In contrast, Genazzani et al. in a study conducted in seven European countries (Belgium, France, Germany, the Netherlands, Spain, Switzerland, and the UK) found climacteric symptoms in 94% of examined respondents [28]. The observed differences between Polish and other European women probably did not result from the difference in the occurrence or severity of climacteric symptoms, but from their subjective declaration in the study. Confirmation of that hypothesis and analysis of its source would probably require further studies.

### 4.3. Depressive Symptoms

In addition, many women exhibit depressive symptoms, both for those who have suffered from depression in the past and for women who have never suffered from depression [29]. Depressive symptoms have been experienced by 32% to even 50% of women during menopause [30], which was also confirmed in our study. In perimenopausal women, the risk of depressive moods is 2–3 times higher than in premenopausal ones [31]. In addition, women with moderate to severe intensity of vasomotor symptoms are more likely to experience severe depressive symptoms [32]. Despite there being no statistical differences between mean age values in both groups, the lower mean age value in the control group could have some influence on the mean age of women reporting depressive symptoms in the B group.

Gibson et al. stated that symptoms of depression are more common in the early perimenopausal period [33], which was also observed in our study, but only for women who did not attend U3A classes. Among U3A participants, depressive symptoms tended to occur later, usually after the age of 55. Perhaps the greater mental and physical activity of women participating in such activities resulted in reduced incidence of depression. We were highly surprised by the fact that among U3A participants, there was a higher percentage of women with moderate and severe depressive symptoms than in the control group. The possible explanation for that phenomenon is that women suffering from depressive symptoms intentionally attend U3A classes to improve their psychological well-being through social relations and contact with specialists involved in health promotion and education. We do believe that further prospective studies are needed to assess whether and how U3A classes affect the symptoms of postmenopausal depression.

### 4.4. Physical Activity

Physical activity is one of the simplest nonpharmacological ways that may reduce the severity of depressive symptoms. More regular physical exercise seems to be particularly beneficial in the climacteric period, when women are exposed to chronic diseases and weight gain that results in increased activity of the adrenergic system, insulin resistance, and hyperinsulinemia. Physical activity makes maintaining or losing weight easier, thus reducing the risk of developing many chronic diseases [34]. The lack of regular exercise is a common problem for people who are advised to increase their activity. It has been proven that internal motivation associated with positive feelings after exercise are the most important factors that may facilitate regular activity. Additionally, daily routine helps patients get used to physical activity and allows them to socialize while exercising in a group. It should be mentioned that menopausal symptoms, however unfavorable, hardly affect the desire to take or decrease physical activity [35]. This observation was also confirmed in our study. We found that mildly and moderately expressed climacteric symptoms do not affect the level of physical activity, which was satisfactory in both groups, although it was not identical. U3A participants were more physically active in the field of work and leisure time, while women in the control group spent a large amount of time in a sedentary position. As the study groups did not differ in professional status, it can be concluded that the mobilizing factor was an opportunity to participate in organized activities and social meetings. The results indicating a lower total physical activity level in the U3A group of participants were surprising, although statistically insignificant, and were due to more frequent car travels.

Similar results were observed by Skrzypulec et al., who showed a high level of physical activity associated with professional work but a lower level during free time [36].

Numerous studies proved that vasomotor symptoms of menopause may increase the risk of depression or depressive moods. It was also documented that increased physical activity may reduce menopausal symptoms, thus reducing the risk of depression [37]. Therefore, aerobic exercises should be strongly recommended to all women during menopause, particularly as they also have a positive effect on both the quality of sleep and intimate life [38,39]. One of the forms of physical activity suggested for seniors in Polish U3A is yoga. It must be noted that yoga is eagerly practiced by women during menopause, as it improves the quality of life, increases the level of serum calcium, and also improves the functioning of the respiratory, muscular, and circulatory systems [40,41].

To summarize, climacteric symptoms and related depression disorders definitely worsen the quality of women’s life. Healthcare professionals must be aware of the importance of those problems and their impact on both patients’ and their families’ life. Encouraging women to undertake physical activity has a great impact on reducing the depressive symptoms and improving well-being. It is also worth remembering that the social environment has a great influence on physical and mental health. Thus, participation in a social group gives a sense of belonging and is a protective factor in the case of depression. More importantly, not only are informal social relations (marriage, friends) important, but also formalized social relations in organizations and associations, such as Universities of the Third Age.

## 5. Limitations

Our study has also some limitations. Firstly, a sample size calculation was not carried out and the conclusion should be taken carefully because the power of the study is not known. Secondly, we did not investigate the hormonal status of the participants and did not perform physical examination. The data about the level of physical activity, climacteric and depressive symptoms, and anthropometrics were based on the self-reported questionnaires and patients’ declarations. Thirdly, questionnaire surveys have their own limitations, such as the superficial responses or quantitative nature of the research, as well as the inability to deepen information in direct contact with the study group. However, these are standard tools that allow diagnosing and evaluating the intensity of a studied variable, regardless of its causes. We did not ask participants about their history of depression, but our intention was to investigate the relationship between the current mood and other parameters of women’s psychosocial functioning. Finally, we did not consider the quality of the participants’ social relations, but only the quantitative indicator. However, it is known that the quality of relationships, both formal and informal, is equally important and even more important than their quantity. For this reason, our study should be treated as an introduction to a more qualitative analysis of social relationships in peri- and postmenopausal women. Therefore, further research will include questions about the time spent on social networking or participation in social activities within three months before the survey is completed. It also seems important to consider further research based on STRAW (Stages of Reproductive Aging Workshop) criteria.

## 6. Conclusions

We conclude that participation in formal social groups is associated with higher level of physical activity related to professional work, leisure time, and sitting time, and reduced severity of depressive symptoms in both peri- and postmenopausal women. Better understanding of the relationship between the physical and psychosocial functioning of women at midlife, participating in any formal groups, can help design prevention programs and support successful aging. In addition, the research serves to screen participants’ health problems and refer them to specialist medical and psychological care.

## Figures and Tables

**Table 1:** Distribution of the groups researched according to sociodemographic variables.

Variables		Group A *N* (%)	Group B *N* (%)	X^2^
Education	High	52 (19.05)	81 (23.28)	2.95
	Secondary	146 (53.48)	173 (49.71)	
	Primary	75 (27.47)	94 (27.01)	
Work	Yes	141 (51.65)	156 (44.83)	2.85
	No	132 (48.35)	192 (55.17)	
Material Status	High	48 (17.58)	51 (14.66)	8.51 *
	Medium	158 (57.88)	192 (55.17)	
	Low	67 (24.55)	105 (30.17)	
Marital Status	Married	220 (80.59)	260 (74.71)	3.03
	Widowed	33 (12.09)	55 (15.80)	
	Divorced	20 (7.33)	33 (9.48)	
Children	Yes	212 (77.66)	222 (63.79)	14.22 **
	No	61 (22.34)	126 (36.21)	

* *p* < 0.05; ** *p* < 0.01 for Chi^2^ (*X*^2^) Test.

**Table 2:** The level of physical activity of women in particular areas according to IPAQ.

Group	Group A	Group B	T-Student
IPAQ Domains	Average ± SD (MET-min/week)	Average ± SD (MET-min/week)	
Job-Related	4780.3 ± 7723.19	2464.89 ± 3485.43	3.66 **
Housework	2138.01 ± 2990.12	1822.2 ± 3047.27	1.29
Transportation	798.29 ± 2050.6	1060.63 ± 2420.41	1.43
Leisure Time	2072.42 ± 2954.84 (60–18,480)	1984.8 ± 3077.32 (0–18,480)	2.66 **
Sitting Time	174.61 ± 138.79	197.83 ± 132.14	2.12 *
Total	3484.2 ± 4871.38	3914.35 ± 5573.89	1.01

* *p* < 0.05; ** *p* < 0.01 for T—Student test; data are mean: SD: standard deviation; IPAQ—International Physical Activity Questionnaire, MET—metabolic equivalent of work.

**Table 3:** Results of Kupperman Index depends on presence or lack of hormonal replacement therapy in study group (group A) and control group (group B).

Group		Climacteric Symptoms			
		Lack	Light	Medium	Severe
Group A	NM *N* = 244	64.81%	12.96%	4.81%	6.67%
	MHT *N* = 9	2.59%	0.37%	0.37%	0.0 %
	PEs *N* = 20	5.19%	1.85/%	0.0 %	0.37%
*X*^2^; df; *p*	5.18; 6; >0.52				
Group B	NM *N* = 275	52.89%	9.25%	8.09%	8.09%
	MHT *N* = 30	6.65%	0.58%	0.87%	0.58%
	PEs *N* = 43	9.25%	2.31%	1.16%	0.29%
*X*^2^; df; *p*	6.23; 6; >0.39				

Chi^2^ (*X*^2^) test; df—degrees of freedom; *p* < 0.05; NM—non-use of medicaments; MHT—menopausal hormone therapy; PEs—phytoestrogens.

**Table 4:** Comparison of the study group (group A) and control group (group B) for symptoms of depression and level of physical activity.

Symptoms of Depression	IPAQ Level	Group A	Group B	*X* ^2^
		*N* (%)	*N* (%)	
Yes	High	42 (15.61)	51 (14.70)	1.63
	Moderate	64 (23.79)	107 (30.84)	
	Low	11 (4.09)	19 (5.48)	
No	High	36 (13.38)	61 (17.58)	11.76 *
	Moderate	108 (40.15)	90 (25.94)	
	Low	8 (2.97)	19 (5.48)	

* *p* < 0.05.
